# Quantification of Carbohydrates in Grape Tissues Using Capillary Zone Electrophoresis

**DOI:** 10.3389/fpls.2016.00818

**Published:** 2016-06-13

**Authors:** Lu Zhao, Ann M. Chanon, Nabanita Chattopadhyay, Imed E. Dami, Joshua J. Blakeslee

**Affiliations:** ^1^Department of Horticulture and Crop Science, Ohio Agricultural Research and Development Center, The Ohio State University, Wooster, OHUSA; ^2^Ohio Agricultural Research and Development Center Metabolite Analysis Cluster, The Ohio State University, Wooster, OHUSA; ^3^Kentucky Tobacco Research and Development Center, University of Kentucky, Lexington, KYUSA

**Keywords:** capillary zone electrophoresis, carbohydrate analysis, monosaccharide separation, raffinose family oligosaccharides, cold stress

## Abstract

Soluble sugars play an important role in freezing tolerance in both herbaceous and woody plants, functioning in both the reduction of freezing-induced dehydration and the cryoprotection of cellular constituents. The quantification of soluble sugars in plant tissues is, therefore, essential in understanding freezing tolerance. While a number of analytical techniques and methods have been used to quantify sugars, most of these are expensive and time-consuming due to complex sample preparation procedures which require the derivatization of the carbohydrates being analyzed. Analysis of soluble sugars using capillary zone electrophoresis (CZE) under alkaline conditions with direct UV detection has previously been used to quantify simple sugars in fruit juices. However, it was unclear whether CZE-based methods could be successfully used to quantify the broader range of sugars present in complex plant extracts. Here, we present the development of an optimized CZE method capable of separating and quantifying mono-, di-, and tri-saccharides isolated from plant tissues. This optimized CZE method employs a column electrolyte buffer containing 130 mM NaOH, pH 13.0, creating a current of 185 μA when a separation voltage of 10 kV is employed. The optimized CZE method provides limits-of-detection (an average of 1.5 ng/μL) for individual carbohydrates comparable or superior to those obtained using gas chromatography–mass spectrometry, and allows resolution of non-structural sugars and cell wall components (structural sugars). The optimized CZE method was successfully used to quantify sugars from grape leaves and buds, and is a robust tool for the quantification of plant sugars found in vegetative and woody tissues. The increased analytical efficiency of this CZE method makes it ideal for use in high-throughput metabolomics studies designed to quantify plant sugars.

## Introduction

Cold or freezing temperatures are a source of abiotic stress that can dramatically inhibit plant growth and development. Freezing temperatures can severely damage crop plants, particularly woody perennials which must over-winter in field conditions. Winter damage can result in bud or meristem death, resulting in decreased productivity the following spring, and a concomitant economic loss to both crop producers and the food industry (Warmund et al., 2008; Dami and Lewis, 2014). Specialty fruit producers, and the grape industry in particular, are at the highest risk of suffering economic losses from cold stress and winter damage. For example, in 2004 a single freezing event caused significant yield decrease to grape producers and led to an estimated loss of $63.6 million to the New York wine industry (Zabadal et al., 2007). The state of Ohio also experienced its greatest loss since 2010 in 2014 due to consecutive, severe freezing events (Dami and Lewis, 2014). In order to survive harsh winters, plants increase their freezing tolerance upon exposure to non-freezing temperatures through cold acclimation (Thomashow, 1999). Over the past two decades, research in the field of winter damage and cold acclimation in crop plants has strongly supported an essential role for soluble sugars, especially raffinose family oligosaccharides (RFOs), in establishing and maintaining freezing tolerance in both herbaceous and woody plants (Castonguay et al., 1995; Jones et al., 1999; Palonen and Junttila, 2002; Grant et al., 2009; Grant and Dami, 2015). RFOs have been proposed to function as osmo- and cryo-protectants during freezing stress, possibly by serving as a water substitute and forming hydrogen bonds with cellular macromolecules (and maintaining protein secondary and tertiary structure); or by interacting with phospholipid headgroups, preserving plasma membrane integrity and cell turgor (Van den Ende, 2013). Accumulations of specific RFOs, such as raffinose, stachyose, and verbascose, in response to cold stimulus, have been reported in *Ajuga reptans*, alfalfa, grape, and red raspberry (Bachmann et al., 1994; Castonguay et al., 1995; Jones et al., 1999; Palonen and Junttila, 2002; Grant et al., 2009). In grape, previous work has demonstrated that increases in the levels of glucose, fructose, sucrose, raffinose, and stachyose present in buds have a strong positive correlation with freezing tolerance (Grant and Dami, 2015). In these studies, it was observed that raffinose levels in grape buds were higher in cold hardy than in cold sensitive grape cultivars; and that raffinose levels in basal buds were higher than those in apical buds (Grant and Dami, 2015). Interestingly, similar patterns of raffinose accumulation have been observed in alfalfa cultivars undergoing cold stress, indicating that accumulation of RFOs may also play an important role in freezing tolerance in non-woody species (Castonguay et al., 1995). Given the putative involvement of soluble sugars in plant cold stress responses, the development of reliable methods for quantifying soluble sugars in herbaceous and woody plant tissues is essential in understanding freezing tolerance.

A number of different analytical platforms have traditionally been employed in carbohydrate analyses. Traditionally, high-pressure liquid chromatography (HPLC) has been used to separate and quantify sugars, particularly higher-order sugars and sugar polymers (Van den Ende et al., 1996; Vendrell-Pascuas et al., 2000). Unfortunately, however, detection of carbohydrates under neutral pH conditions, which are not chromophores or fluorophores, following separation via HPLC, is challenging as it must rely on simple UV absorbance of the sugar molecules (Montero et al., 2004). To overcome this challenge, most HPLC-based methods use either a refractive index (RI) detector or an evaporative light scattering (ELS) detection system, in which the sample emerging from the HPLC system is rendered into vapor phase using nitrogen gas and then passed through a light-scattering detection system capable of detecting the disruption of a light beam by sugar molecules, instead of a UV/visible photodiode array to quantify sugar molecules (Raessler, 2011; Li et al., 2013; Oliver et al., 2013). However, both RI and ELS detection systems exhibit maximum sensitivity under aqueous conditions (Montero et al., 2004) and do not permit the use of common HPLC solvents, such as acetonitrile. Because of this, methods using RI or ELS are limited in column selection to ion-exchange (IE)/size-exclusion chromatography (SEC) columns which function well when an isocratic flow of water is used as the buffer system for sugar separations. These columns are therefore often optimized only for the separation of a single class of sugars. For example, columns optimized for the separation of monosaccharides usually do not resolve oligosaccharides well, and vice-versa, making it difficult to design an HPLC method capable of separating the broad range of carbohydrates found in plant tissues. These drawbacks have led to the development of alternative methods to identify and quantify plant sugars (Colon et al., 1993; Webb et al., 1995; Cérantola et al., 2004; Montero et al., 2004). ^1^H-Nuclear magnetic resonance (NMR) is an example of one of these alternative methods, and has been used to determine the structures of carbohydrates accumulating in plant tissues during cold stresses (Dai et al., 2010; Nair et al., 2012; dos Santos et al., 2013). While it has been able to provide high resolution analysis of sugar structure, this technique requires expensive instrumentation, and the individual NMR measurements require a large amount of pure sample (either 1 g of lyophilized tissue or 3 mg of purified oligosaccharide; Tuomivaara et al., 2015). Alternatively, gas-chromatography coupled with mass spectrometry (GC–MS) has a relatively low LOD, is able to resolve many soluble sugars, and allows spectroscopic identification of sugar moieties (Gibeaut and Carpita, 1991; Webb et al., 1995; Montero et al., 2004; Schliemann et al., 2008; Gao et al., 2015). As a result, GC–MS has become the method of choice for in-depth identification and quantification of carbohydrates in plant tissues (Grant et al., 2009; Ruiz-Matute et al., 2011; Dhuli et al., 2014; Grant and Dami, 2015; Hu et al., 2015). However, carbohydrate analysis by GC–MS is somewhat difficult to employ in high-throughput metabolomics studies, or in studies where only small amounts of tissue are available, as this method generally requires the use of a multi-step derivatization process (necessary to ensure that individual sugars interact with the GC column matrix), which employs toxic reagents and in which sample loss inevitably occurs at each step of the process (Ruiz-Matute et al., 2011). These limitations have made it difficult to use standard GC–MS methods in high-throughput metabolomics studies designed to profile short- and long-term changes in sugar levels following cold stress, and have driven efforts to develop alternate, cost-effective, and “derivatization-free” methods to quantify carbohydrates in plant samples.

Capillary zone electrophoresis (CZE) provides a potential alternate method by which soluble carbohydrates from plant samples can be quantified. In CZE, molecules injected into the capillary-buffer system migrate in an electrophoretic field, in which the rate of movement of individual molecules through the capillary and detector (usually a photo-diode array) is based on their charge-to-mass ratio (Oefner and Chiesa, 1994). CZE has previously been used to successfully separate and quantify several different sugars in carbohydrate standard mixtures, food extracts, and fruit juices (Hoffstetterkuhn et al., 1991; Vorndran et al., 1992; Colon et al., 1993; Lu and Cassidy, 1993; O’Shea et al., 1993; Honda, 1996; Paulus and Klockow, 1996; Bazzanella and Bachmann, 1998; Rovio et al., 2007). Most notably for the analysis of sugars in plant samples, a CZE method to separate simple sugars in fruit juices has recently been developed under alkaline conditions, using direct UV detection of carbohydrates at a wavelength of 270 nm (Rovio et al., 2007). In this method, neutral carbohydrates are converted into sugar acids through the use of an alkaline sodium hydroxide/phosphate buffer system (pH 12.6), separated via CZE, and detected at a UV absorbance of 270 nm (Rovio et al., 2007). While this method was successful in separating the sugars found in fruit juices, it should be noted that fruit juices, particularly those from citrus fruits, generally have a relatively simple carbohydrate composition, consisting primarily (or entirely) of sucrose, glucose, and fructose (Rovio et al., 2007; Jiang et al., 2015). To date, neither CZE methods developed to analyze fruit juice samples, nor those developed to analyze other carbohydrate mixtures, have successfully been used to separate the broader range of sugars present in the complex matrix of most plant extracts. Additionally, it was unclear whether CZE methods could be used to profile the longer-chain oligosaccharides described above (i.e., RFOs like raffinose, stachyose, etc.) which function in plant stress responses. Here, we present an optimized CZE method capable of separating and quantifying mono-, di-, tri-, and tetra-saccharides isolated from both herbaceous and woody samples, using grape leaves as a model plant tissue. The CZE method presented here does not require the use of specialized solvents, or derivatization of the samples, and exhibits an analytical efficiency comparable to (or, for some sugars, greater than) GC–MS methods, making it an ideal method for use in high-throughput metabolomics studies designed to quantify the accumulation of plant sugars throughout cold-stress responses.

## Materials and Methods

### Chemicals and Reagents

Glucose, fructose, sucrose, *myo*-inositol, mannose, xylose, ribose, lactose, arabinose, and galactose were purchased from Sigma-Aldrich (St. Louis, MO, USA). Galactinol, ribitol, and trehalose were supplied by Santa Cruz Biotechnology (Santa Cruz, CA, USA). Raffinose and cellobiose were obtained from Fluka (Sigma-Aldrich, St. Louis, MO, USA) and Supelco (Sigma-Aldrich, St. Louis, MO, USA), respectively. Stachyose was from TCI America (Tokyo, Japan). All standard reagents were of analytical grade. Standard sugar solutions were prepared by dissolving individual sugars in ultra-pure (liquid chromatography mass spectrometry-grade, 18 MΩ) water (Fisher Scientific, Pittsburgh, PA, USA). The concentration of stock solution for each sugar component was either 1 or 10 mg/mL. Chemical reagents for derivatization prior to GC–MS analyses, including pyridine, phenyl-β-D-glucopyranoside, hydroxylamine hydrochloride, hexamethyldisilazane (HMDS) and trifluoroacetic acid (TFA) were purchased from Sigma-Aldrich (St. Louis, MO, USA).

### Generation of Calibration Curves, and Determination of Limit of Detection (LOD) and Limit of Quantification (LOQ)

To generate calibration curves, 10 concentrations of each individual sugar component (2.5, 5, 10, 25, 50, 100, 200, 300, 400, and 500 ng/μL) were subjected to CZE analysis. Briefly, stock solutions of carbohydrate calibration standards (*myo*-inositol, galatinol, stachyose, raffinose, sucrose, lactose [used as an I-STD in CZE analysis], trehalose, cellobiose, galactose, glucose, mannose, fructose, arabinose, xylose, and ribose) were diluted with ultra-pure water. Each sample was passed through a 0.2 μm nylon filter (Phenomenex, Inc., Torrance, CA, USA) prior to injection on the CZE system. To determine the LOD and LOQ, stock solutions (1 mg/mL of each sugar) were diluted in ultra-pure water to 2.5, 5, 10, 15, 20, and 25 ng/μL (for ribose), and 0.5, 1, 2, 3, 4, and 5 ng/μL (for the remaining 14 sugars), filtered as described above, and analyzed via CZE. LOD and LOQ were determined by monitoring UV absorbance peaks and background absorbance for each dilution of the individual sugar standards. The LOD threshold was defined as the lowest concentration of a sugar generating a peak with a signal-to-noise ratio of greater than 3, and the LOQ as the lowest concentration of a sugar generating a peak with a signal-to-noise ratio of greater than 10. Three technical replicates (individual dilutions) were performed for each concentration measurement, and each replicate was analyzed (i.e., injected onto the CZE system) twice. For GC–MS analyses, 12 concentrations of each individual carbohydrate were measured: glucose, fructose, and sucrose (2, 50, 100, 200, 250, 300, 350, 400, 500, 600, 700, and 800 ng/μL); *myo*-inositol (1, 10, 50, 100, 125, 150, 175, 200, 250, 275, 300, and 350 ng/μL); galactinol (1, 3, 5, 10, 20, 30, 40, 50, 60, 80, 100, and 120 ng/μL); raffinose (3, 5, 10, 20, 50, 60, 80, 100, 120, 150, 180, and 200 ng/μL); stachyose (3, 5, 10, 15, 20, 30, 40, 50, 60, 80, 100, and 120 ng/μL); ribitol (3, 5, 10, 20, 50, 100, 150, 200, 250, 300, 350, and 400 ng/μL). Prior to GC–MS analyses, dilutions were subjected to derivatization, as described below. LOD and LOQ were determined as described in the CZE section above, with the exception that the total ion count (TIC) peaks generated by the mass spectrometer (electron impact detector) for each sugar, rather than UV absorbance peaks, were employed in signal-to-noise calculations. For GC–MS studies, three technical replicates (dilutions and derivatizations) were performed for each concentration analyzed, and each replicate was analyzed (i.e., injected into the GC–MS system) twice.

### Plant Materials

Cabernet franc (CF, *Vitis vinifera*) grapevines were grown at Horticulture Research Unit 2, Ohio Agricultural Research and Development Center (OARDC), Wooster, OH (lat. 40°47′ N; long. 81°55′ W). Grape leaves were harvested on October 17, 2014, and immediately flash-frozen in liquid nitrogen. Frozen samples were transported to the laboratory on dry ice and then stored at -80°C until analysis.

### Sugar Extractions

Frozen leaves were lyophilized using a FreeZone Plus 12 Liter Cascade Console Freeze Dry System (Labconco Corp., Kansas City, MO, USA), and triple ground in a mortar and pestle using liquid nitrogen. For CZE analyses, an aliquot (45 mg) of leaf powder was then suspended in 200 μL of solvent and incubated on an nutator (Thomas Scientific, model: BioMixer, Swedesboro, NJ, USA) for 1 h at 4°C. Extraction solvents used in the course of the study included: 80% ethanol, 75% ethanol, 60% ethanol, and water. Extraction buffers contained lactose at a concentration of 250 ng/μL as an I-STD. Following incubation, samples were centrifuged at 12,000 × *g* for 15 min at 4°C, supernatants were transferred to 1.7 mL microcentrifuge tubes, and samples were stored on ice for immediate CZE analysis. Each aliquot of tissue was extracted four times (for a total extraction volume of 800 μL per 45 mg tissue), and the extracts were pooled and dried under a stream of nitrogen gas, then resuspended in 1 mL ultra-pure water and filtered prior to CZE analysis. Three technical replicates (individual extracts) were performed for each extraction solvent, and each replicate was analyzed (i.e., injected onto the CZE system) twice.

For sugar quantification by GC–MS, grape leaf samples were triple ground in liquid nitrogen as described above, with the following modifications. Aliquots of tissue (20 mg) were extracted using 200 μL of 75% ethanol, containing 250 ng/μL of ribitol as an I-STD. Each aliquot of sample was solvent extracted three times (for a total volume of 600 μL per 20 mg tissue), and the extracts were pooled and dried under nitrogen gas. The dried extracts were subjected to derivatization as described below prior to GC–MS analysis (Streeter and Strimbu, 1998; Grant et al., 2009; Grant and Dami, 2015). Three technical replicates (individual extracts) were performed, and each replicate was analyzed (i.e., injected into the GC–MS system) twice.

### Separation and Quantification of Soluble Sugars by CZE

Capillary zone electrophoresis-based separation of sugars was carried out using a P/ACE MDQ Capillary Electrophoresis system (Beckman Coulter, Fullerton, CA, USA). Sugars were detected using a diode array detector (DAD) set to measure absorbance at a wavelength of 270 nm, with a 10 nm band width. Sugars were resolved using a fused silica capillary (part # 338454, AB Sciex, Redwood City, CA, USA), with a length of 70 cm (50 cm effective length), an internal diameter of 75 μm, and an external diameter of 375 μm. The CZE was operated using Beckman Coulter 32 Karat software Version 8.0 (Beckman Coulter). As previous studies (Rovio et al., 2007) have demonstrated that fructose, sucrose, and glucose can be resolved with CZE using an alkaline buffer system, a series of basic electrolyte buffers were assayed for use as CZE buffers. In initial trials, alkaline running buffers were prepared by combining a stock solution of 1 M sodium hydroxide (NaOH) and a stock solution of 500 mM disodium hydrogen phosphate (Na_2_HPO_4_) for a final concentration of 130 mM NaOH and 36 mM Na_2_HPO_4_, after which the pH was adjusted to 12.60 ± 0.02, 12.80 ± 0.02, or 13.00 ± 0.02 via the dropwise addition of 1 M HCl. Alkaline running buffers consisting of 130 mM NaOH (without Na_2_HPO_4_), pH 12.90 ± 0.02, 13.00 ± 0.02, or 13.20 ± 0.02, were also investigated during studies designed to optimize resolution of individual sugars. The pH values of all electrolyte/running buffers were confirmed using a pH meter (Jenway model 3510, Essex, UK).

Capillary zone electrophoresis running conditions were modified from those initially published by Rovio et al. (2007). Both the capillary and the samples being analyzed, were maintained at 15°C. New capillaries were conditioned using the following protocol (unless noted, all steps used a pressure of 20 psi): 1 M NaOH for 5 min, 5 min of equilibration (no flow), 1 M NaOH for 5 min, H_2_O for 5 min. After each change of background electrolyte (i.e., when a running buffer of a different pH was applied), the capillary was re-conditioned using the following protocol (unless noted, all steps used a pressure of 20 psi): 1 M NaOH for 1 min, H_2_O for 1 min, alkaline column electrolyte buffer (pH 12.6, 12.8 13.0, or 13.2) for 1 min. Following these rinses, the separation voltage was raised linearly from 0 to 10 kV over the course of 2 min and maintained at 10 kV for 10 min. The column was then rinsed with 1 M NaOH for 2 min, then H_2_O for 2 min (each at 20 psi). The conditioning method above was repeated until a stable baseline absorbance (measured using a UV wavelength of 270 nm) and current were both observed.

For sample measurements, the capillary was rinsed with alkaline electrolyte buffer (pH 12.6, 12.8 13.0, or 13.2) at 20 psi for 2 min immediately prior to each sample injection. Samples were injected using 0.5 psi for 5 s, followed by the injection of a plug of alkaline electrolyte buffer at 0.1 psi for 10 s. Samples were separated in alkaline electrolyte buffer using a range of separation voltages. Separation voltages were ramped linearly from 0 to either 8, 10, 12, 14, or 16 kV over 2 min, followed by a separation time of 75 min under normal polarity (i.e., the cathode in the destination vial and the anode in the source vial). After every sample run, the capillary was washed using the following steps (all steps used a pressure of 20 psi): 10% v/v acetic acid for 4 min, H_2_O for 3 min, and alkaline electrolyte solution for 3 min. The column was re-conditioned as described above with fresh running buffer after every 10 runs.

### Derivatization of Sugars for GC–MS Analyses

Sugars were derivatized for downstream GC–MS analyses as described previously (Streeter and Strimbu, 1998), with some modifications. Briefly, sugars were extracted in 75% ethanol, transferred into target vials, and dried under nitrogen gas, as described above. Sugar standard samples were then resuspended in 155 μL of pyridine and 125 μL of STOX solution (25 mL pyridine, 25 mg/mL hydroxylamine hydrochloride and 0.8 mg/mL phenyl-β-D-glucopyranoside; Streeter and Strimbu, 1998). Samples were then vortexed for 10 s and incubated in Reacti-Therm Heating Modules (model 18935, Pierce, Rockford, IL, USA) at 70°C for 40 min. Following this incubation, vials were allowed to cool to room temperature (approximately 22°C); 200 μL of HMDS and 20 μL of trifluoroacetic acid (TFA) were then added to each vial, and samples were vortexed for 10 s. Samples were then placed at 4°C overnight (12 h) to allow sediments to precipitate, after which supernatants were transferred to 1.5 mL target vials for GC–MS analysis. Carbohydrates present in leaf extracts were derivatized using the procedure described above, with the following modifications: 310 μL of pyridine and 250 μL of STOX solution were used to resuspend dried samples; and 400 μL of HMDS and 40 μL of TFA were used to derivatize leaf extracts.

### Separation and Quantitation of Soluble Sugars by GC–MS

Derivatized sugars (from carbohydrate standard mixtures or grape leaf extracts) were analyzed as described previously (Streeter and Strimbu, 1998; Grant et al., 2009; Grant and Dami, 2015), with the following modifications. Samples were injected onto an Agilent 6890 gas chromatograph equipped with a 30 m HP-5 cross-linked (5%-Phenyl)-Methylpolysiloxane column (HP5-MS, 250 μM inner diameter; 0.25 μm outer diameter) linked to an Agilent 5973 mass spectrometer (Agilent Technologies, Santa Clara, CA, USA). The inlet temperature on the GC was set at 280°C, and sugars were separated using helium as the carrier gas (1 mL/min) and the following thermal gradient: 180°C for 2 min, 6°C/min to 220°C, hold for 2 min, and 20°C/min to either 300 or 320°C, hold for 30 min (Streeter and Strimbu, 1998; Grant et al., 2009; Grant and Dami, 2015). The GC–MS system was operated using the Agilent MSD ChemStation Software (Agilent Technologies, Santa Clara, CA, USA). Mass spectrometry (MS) settings were: MS transfer line 280°C, ion source 230°C, electron impact energy 70 eV, with a scan rate of 2 scan/sec. from 50 to 800 amu. Mass spectra were analyzed using Agilent MassHunter software (Agilent Technologies, Santa Clara, CA, USA). Derivatized sugars were identified by comparison of retention times and mass spectra to those of authentic standards, and quantified using standard curves generated from each authentic standard. Retention times for sugars measured were: 4.92 min (ribitol, [I-STD]), 7.82 min (fructose), 8.48 min (glucose), 9.81 min (*myo*-inositol), 15.03 min (sucrose), 16.66 min (galactinol), 19.28 min (raffinose), and 36.75 min (stachyose; **Table 3**).

### Statistical Analysis

For CZE analyses, data collection (i.e., retention time, peak area, and CZE chromatographs) was performed using the 32 Karat 8.0 software package (Beckman Coulter). For GC–MS analyses, data collection (i.e., retention time, peak area and GC–MS chromatographs) was performed using ACD MS Manager with IntelliXtract (ACD Spectrus MS Workbook Suite V2012). Both CZE and GC–MS data were subjected to paired *t*-test using Minitab statistical software (Minitab, Inc., State College, PA, USA). Significant differences were claimed at *P* < 0.05.

## Results

### Preliminary CZE Separation of Complex Mixtures of Plant Carbohydrates Using Alkaline Buffer Conditions

**FIGURE 1:**
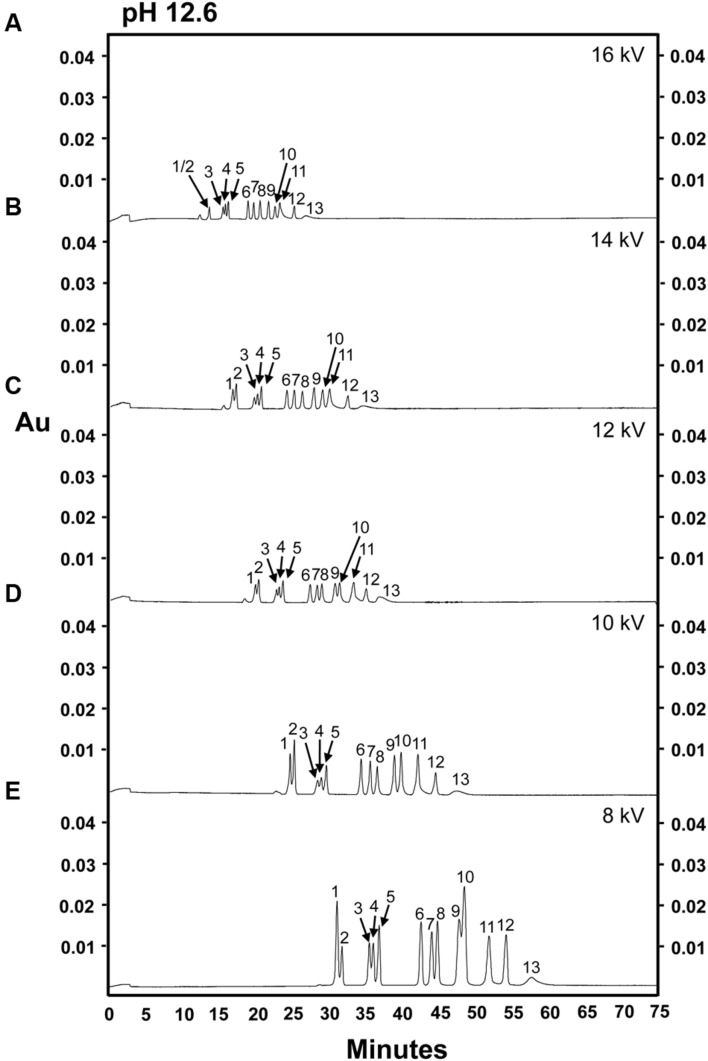
**Separation of 14 carbohydrates in an electrolyte solution of pH 12.6 at different separation voltages. 100 ng of each sugar was injected onto the column. (A)** 16 kV. **(B)** 14 kV. **(C)** 12 kV. **(D)** 10 kV. **(E)** 8 kV. Peak identities: (1) *myo*-inositol; (2) galactinol; (3) stachyose; (4) raffinose; (5) sucrose; (6) lactose (I-STD); (7) cellobiose; (8) galactose; (9) glucose; (10) mannose; (11) fructose/arabinose; (12) xylose; (13) ribose. Electrolyte solution: 130 mM NaOH and 36 mM Na_2_HPO_4_. Experiment repeated three times, representative data shown here.

A sugar mixture including 13 sugars commonly found in plant samples, including both structural and non-structural sugars (glucose, sucrose, fructose, *myo-*inositol, galactinol, stachyose, raffinose, cellobiose, galactose, mannose, arabinose, xylose, and ribose) and lactose (I-STD) was prepared and separated using an alkaline electrolyte solution of pH 12.6 at 16 kV as described by Rovio et al. (2007; **Figure 1A**). When this separation was attempted, a current of 230 μA, much higher than the 140 μA encountered in the fruit juice separations reported in Rovio et al. (2007), was generated across the capillary circuit. Interestingly, the current generated across the capillary increased approximately 5 μA with each consecutive sample run, and rapidly approached the system limit of 300 μA. Consistent with the increases in current observed as samples were run (which indicated resistance across the circuit), we observed a simultaneous increase in the temperature generated across the capillary. While, this could be at least partially buffered by the column cooling system of the P/ACE MDQ CZE unit, the increased temperatures observed resulted in an approximately 20–25% decrease in capillary life. Despite these difficulties, we were able to achieve at least partial separation of the carbohydrate mixture using this method (**Figure 1A**). However, several sugars were not cleanly separated using this method, with the most prominent clusters of unresolved sugars occurring at approximately 13 min (*myo*-inositol and galactinol [peak 1/2]), 16 min (stachyose, raffinose, and sucrose; peaks 3–5, respectively) and 23 min (mannose [peak 10] and fructose and arabinose [peak 11]) into the run (**Figure 1A**). In an effort to simultaneously decrease the current generated across the capillary and increase the resolution of the *myo*-inositol-galactinol, stachyose-raffinose-sucrose and mannose-fructose-arabinose peaks, samples were re-analyzed using the same alkaline electrolyte buffer (pH 12.6), but a decreased voltage of 14 kV (**Figure 1B**). While this allowed increased separation of mannose from the combined fructose/arabinose peak, it did not enhance the efficiency of the method, as the poor resolution of stachyose, raffinose and sucrose was unchanged. Additionally, running at the lower voltage caused *myo*-inositol (peak 1) and galactinol (peak 2) to co-elute, and fructose and arabinose co-eluted as a single peak with a tail-like shoulder (peak 11). **Table 1** shows the calibration curves, LOD and LOQ of the 14 carbohydrate assays, as well as lactose, which was used as an I-STD when sugars were separated in an alkaline electrolyte buffer of pH 12.6 at 14 kV. The concentration-response curve showed poor linearity for most of the sugar components tested, with almost all carbohydrates examined yielding coefficients of determination (*R*^2^-values) of less than 0.95 (except for raffinose [*R*^2^ = 0.992] and cellobiose [*R*^2^ = 0.979]). The LOD obtained using this method ranged between 3 and 4 ng/μL for all sugars tested except for ribose, fructose, and arabinose; while the LOQ was between 4 and 5 ng/μL for all sugars tested, again with the exception of ribose, fructose, and arabinose. Ribose was more difficult to detect using this method, and the LOD and LOQ values for this sugar were 25 and 50 ng/μL, respectively. As fructose and arabinose co-eluted, it was impossible to measure either of these sugars accurately, making it impossible to plot concentration-response curves or determine LOD or LOQ for these carbohydrates using the CZE methods described above.

**Table 1:** Linearity, precision, mobility, LOD, and LOQ of sugar components using an alkaline electrolyte buffer of pH 12.6 at 14 kV.

Sugar component	Range (ng/μL)	Calibration curve	*R*-squared	Migration time	Retention index^a^	LOD (ng/μL)	LOQ (ng/μL)
*Myo*-inositol	2.5–500	y = 447.86x + 4350.9	0.846	17.147	0.705	4	5
Galactinol	2.5–500	y = 417.98x + 7768.5	0.897	17.587	0.723	4	5
Stachyose	2.5–500	y = 202.48x + 9259	0.918	19.975	0.821	4	5
Raffinose	2.5–500	y = 467.14x + 2341.6	0.992	20.396	0.838	4	5
Sucrose	2.5–500	y = 331.64x + 11841	0.910	20.883	0.858	3	5
Lactose (ISTD)	2.5–500	y = 957.72x - 12069	0.973	24.339	1	3	5
Cellobiose	2.5–500	y = 861.59x - 8203.2	0.979	25.311	1.040	3	4
Galactose	2.5–500	y = 1232x - 28017	0.936	26.286	1.080	3	5
Glucose	2.5–500	y = 479.68x + 9425.8	0.921	27.843	1.143	3	5
Mannose	2.5–500	y = 1905x - 74249	0.765	28.906	1.187	3	5
Fructose/arabinose^b^	2.5–500	y = 1825.9x - 43997	0.944	30.006	1.232	NA^c^	NA^c^
Xylose	2.5–500	y = 1087.9x - 29482	0.908	32.325	1.327	3	5
Ribose	25–500	y = 956.32x - 51817	0.926	35.577	1.432	25	50

*Sugars were separated using CZE, with an electrolyte buffer of pH 12.6, at 14 kV, as described in Section “Materials and Methods.” A mix of 14 sugar standards was injected onto the P/ACE MDQ CE system. *R*-squared values, LOD, and LOQ were calculated as described in Section “Materials and Methods.” Experiment was repeated three times, representative data from one experimental replicate presented here.*

*^a^Retention index = the retention time of each sugar component divided by the retention time of the I-STD; RI = Rt_*sugar*_/Rt_I-*STD*_.*

*^b^Fructose and arabinose co-elute as a single peak.*

*^c^NA, not applicable (could not be calculated due to co-elution).*

### Optimization of CZE pH and Voltage Conditions

As several sugars co-eluted through the capillary at 14 kV, we hypothesized that reducing the electrophoretic mobility of the sugar by decreasing the separation voltage (Colon et al., 1993) might assist the separation of carbohydrates that had poor baseline resolution (i.e., mannose, fructose, and arabinose) or that tended to co-elute. To investigate this possibility, we performed separations at lower voltages (12, 10, or 8 kV) while maintaining the pH of the electrolyte buffer at 12.6 (**Figures 1C–E**). The migration time of each sugar component was, as expected, increased as the separation voltage decreased (i.e., migration time was inversely proportional to separation voltage), most likely due to decreases in the rate of electro-osmotic flow as the voltage was reduced. Interestingly, although the overall peak separation patterns were consistent over different separation voltages, the peak separation of glucose (peak 9) and mannose (peak 10) was poorer at 8 kV (**Figure 1E**). The use of a separation voltage of 8 kV resulted in the largest peak areas for most sugar components (followed by 10 kV), with the exception of ribose, which exhibited stochastic differences in peak areas at different separation voltages. For all sugars tested, no significant difference in peak areas was observed between running conditions of either 12 or 14 kV.

**FIGURE 2:**
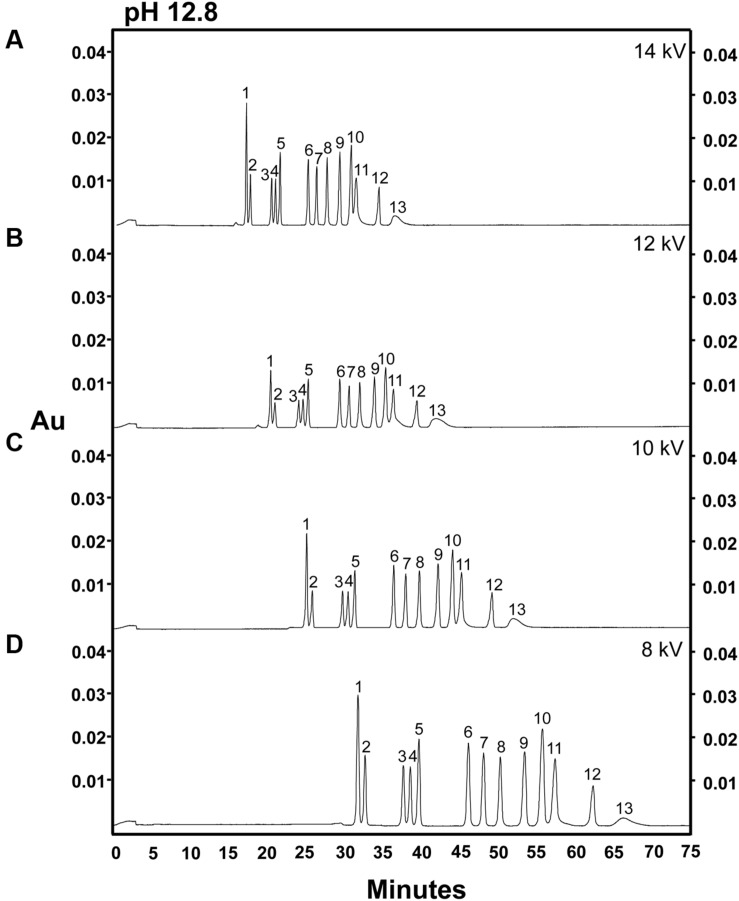
**Analysis of 14 carbohydrates in an electrolyte solution of pH 12.8 at different separation voltages.** 100 ng of each sugar was injected onto the column. **(A)** 14 kV. **(B)** 12 kV. **(C)** 10 kV. **(D)** 8 kV. Peak identities: (1) *myo*-inositol; (2) galactinol; (3) stachyose; (4) raffinose; (5) sucrose; (6) lactose (I-STD); (7) cellobiose; (8) galactose; (9) glucose; (10) mannose; (11) fructose/arabinose; (12) xylose; (13) ribose. Electrolyte solution: 130 mM NaOH and 36 mM Na_2_HPO_4_. Experiment repeated three times, representative data shown here.

Many carbohydrates which are neutral in aqueous solutions at pH 7 are weak acids which will lose one or more protons (usually from a terminal carboxyl group) and become ionized in strongly alkaline solutions (Vorndran et al., 1992; Honda, 1996; Paulus and Klockow, 1996; Rovio et al., 2007). Achieving maximum ionization of all weakly acidic sugar moieties present in a sample is essential for efficient analysis of carbohydrates using CZE, as this method separates compounds via a combination of electrophoretic movement and electro-osmotic flow (i.e., the movement of charged molecules and a charged buffer solution in an electric field applied across the capillary); and, as a result, the speed at which a molecule moves through the capillary system is directly proportional to its charge-to-mass ratio (Colon et al., 1993; Rovio et al., 2007). Because of this, we hypothesized that increasing the pH of the alkaline electrolyte solution used in the CZE system might enhance the ionization of carbohydrates and improve the separation by decreasing the peak width for each carbohydrate species. To test this hypothesis, a 14-sugar mixture was separated at the different separation voltages described above (14, 12, 10, or 8 kV), using alkaline electrolyte buffer of pH 12.8 (**Figure 2**). When the pH of the alkaline electrolyte buffer raised from 12.6 to 12.8, the resolution of individual sugar peaks was enhanced, and we were able to achieve baseline resolution of most of the sugar standards analyzed (**Figure 2**). Moreover, use of an electrolyte buffer of pH 12.8 gave rise to significantly larger peak areas for all sugar components than were observed when an electrolyte buffer of pH 12.6 was used, a phenomenon that held true across at all tested voltages. However, even when the pH of the electrolyte solution was increased to 12.8, fructose and arabinose still co-eluted (peak 11). As observed at pH 12.6, at pH 12.8, significantly larger peak areas for sugars were observed at 8 kV than at higher voltages, again with the exception of ribose (**Figure 2D**). However, there were no differences in peak areas when higher voltages (i.e., 14, 12, and 10 kV) were applied (**Figures 2A–C**). Although, lower voltages (8 kV) increased peak areas for most sugars, the increased pH of electrolyte buffer, rather than the modifications to the separation voltages, had the largest effect on the overall separation and resolution of individual sugar components (**Figures 1** and **2**).

**FIGURE 3:**
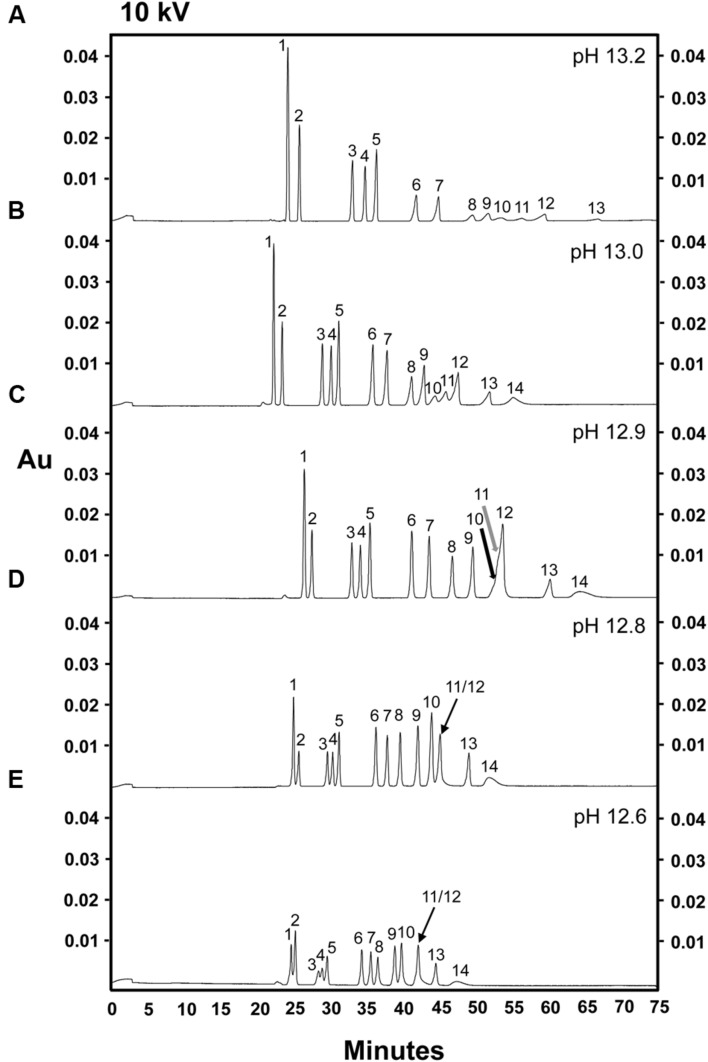
**Comparison of five alkaline electrolytes in the separation of 14 carbohydrates, 100 ng of each sugar component.** Separation voltage: 10 kV. Compared electrolytes, separation current and pH of the electrolyte during analysis. **(A)** 130 mM NaOH (240 μA, pH 13.2). **(B)** 130 mM NaOH (185 μA, pH 13.0). **(C)** 130 mM NaOH (170 μA, pH 12.9). **(D)** 130 mM NaOH-36 mM Na_2_HPO_4_ (150 μA, pH 12.8). **(E)** 130 mM NaOH-36 mM Na_2_HPO_4_ (130 μA, pH 12.6). Peak identities: (1) *myo*-inositol; (2) galactinol; (3) stachyose; (4) raffinose; (5) sucrose; (6) lactose (I-STD); (7) cellobiose; (8) galactose; (9) glucose; (10) mannose; (11) fructose; (12) arabinose; (13) xylose; (14) ribose. Black and gray arrows show the potential peaks of mannose and fructose, respectively. Experiment repeated three times, representative data shown here.

Based on the dramatic increase in sugar resolution observed when the pH of the electrolyte buffer used in our CZE separations was raised from 12.6 to 12.8, we suspected that further increasing the pH might allow the separation of fructose and arabinose, which still co-eluted at pH 12.8. Alkaline electrolyte buffers (this time consisting of only 130 mM NaOH) at pH 12.9, 13.0, or 13.2 were evaluated for their efficiency in separating a complex sugar mixture (**Figures 3A–C**). 10 kV was selected as the initial separation voltage, as this voltage allowed for both good resolution and a moderately fast run time at pH 12.6 and 12.8 (**Figures 1** and **2**). Unexpectedly, the mannose, fructose, and arabinose peaks merged into one large, multi-sugar peak at pH 12.9, although this peak did exhibit two tiny bumps, which likely represent mannose and fructose (**Figure 3C**, black and gray arrows, respectively). At a pH of 13.0, mannose (peak 10), fructose (peak 11) and arabinose (peak 12) were, once again, completely separated (i.e., resolution was achieved between the mannose, fructose, and arabinose peaks; **Figure 3B**). This pH resulted in the best resolution between the sugars present in the 14 sugar standard mixture (**Figure 3**). When the pH was further increased to 13.2, however, the resolution between galactose, glucose, mannose, fructose, arabinose, and xylose (peaks 8–13, respectively) dramatically decreased, and ribose could no longer be clearly detected (**Figure 3A**). The loss of resolution of these monosaccharides may be due to sample degradation resulting from the heat generated across the circuit when the pH 13.2 buffer system was used. The high current (240 μA) observed across the circuit during these runs tends to support this hypothesis. The peak areas observed for *myo*-inositol, galactinol, stachyose, raffinose, and sucrose were consistent at pH 12.8 and 13.0 (**Figures 3B,D**), but were higher at pH 13.2 (**Figure 3A**). As noted above, the electrolyte buffer of pH 13.0, containing 130 mM NaOH, successfully separated soluble sugars from cell wall components (i.e., individual peaks of mannose, fructose, and arabinose), and achieved baseline resolution of almost all sugar components. The only drawback to the separations performed at pH 13.0 was that the peak areas observed for cellobiose, galactose, xylose, and ribose were smaller than those seen when a buffer system of pH of 12.8 was used. In an attempt to improve resolution of sugars at pH 13.0, we modified the buffer system and employed an electrolyte buffer of 130 mM NaOH and 36 mM Na_2_HPO_4_, pH 13.0 (data not shown). The resolutions of the glucose, mannose, fructose, arabinose, xylose, and ribose peaks were poorer in this system (data not shown), suggesting that, consistent with previously published data (Rovio et al., 2007), the addition of disodium hydrogen phosphate did not enhance the sensitivity in the separation of mannose, fructose, and arabinose.

### Separation of Complex Mixtures of Plant Carbohydrates Using an Optimized CZE Method

**FIGURE 4:**
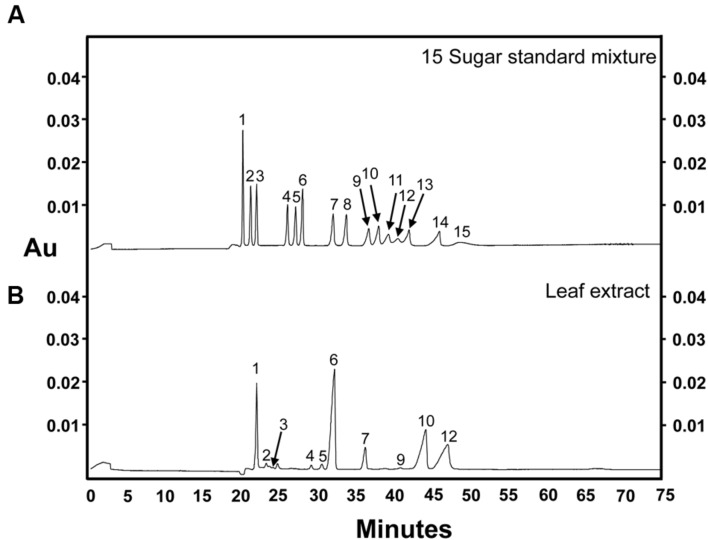
**Sugar separation using the optimized CZE method. (A)** 15-sugar mixture (100 ng of each sugar component) and **(B)** grape leaf extract (two times dilution of 75% ethanol extract). Peak identities: (1) *myo*-inositol; (2) galactinol; (3) trehalose; (4) stachyose; (5) raffinose; (6) sucrose; (7) lactose (I-STD); (8) cellobiose; (9) galactose; (10) glucose; (11) mannose; (12) fructose; (13) arabinose; (14) xylose; (15) ribose. Separation voltage: 10 kV. Electrolyte solution: 130 mM NaOH (185 μA, pH 13). Experiment repeated three times, representative data shown here.

As described above, our data indicated that the optimal alkaline electrolyte buffer system for the separation of sugars from plant extracts consisted of 130 mM NaOH, pH 13.0, which created a current of 185 μA when a separation voltage of 10 kV was applied across the circuit. Calibration curves of sugar standards over a concentration range from 2.5 to 500 ng/μL were generated under these conditions, and are shown in **Table 2**. Trehalose was also included in the sugar mixture to expand the sugar profile generated from plant samples. The standard curves for each sugar showed good linearity across the concentration range tested, with *R*^2^-values of approximately 0.990 for most components except galactose (0.970), glucose (0.978), and ribose (0.950). Although, the migration time for each sugar was slightly longer than the original method attempted (pH 12.6; **Figure 1A**), this was compensated for by the fact that the optimized method resulted in well-defined and symmetrical peaks for most sugar standards and provided the best resolution of mannose, fructose, and arabinose (peaks 11–13, respectively) out of all of the separation conditions tested (**Figure 4A**). The optimized CZE method, using a buffer system of pH 13.0 and a separation voltage of 10 kV, gave an average LOD and LOQ of 1.5 and 3 ng/μL, respectively, for most sugars. The LOD and LOQ values for mannose, fructose and arabinose were 2 and 4 ng/μL, respectively. Although, the LOD and LOQ of ribose (15 and 25 ng/μL) were not as low as those observed for other analyzed sugar standards, at pH 13.0, 10 kV, these values were the lowest achieved in all tested conditions.

**Table 2:** Linearity, precision, mobility, LOD, and LOQ of sugar components using an alkaline electrolyte buffer of pH 13.0 at 10 kV.

Sugar component	Range (ng/μL)	Calibration curve	*R*-squared	Migration time	Retention index^a^	LOD (ng/μL)	LOQ (ng/μL)
*Myo*-inositol	2.5–500	y = 3068.3x - 47674	0.982	21.227	0.626	1	2
Galactinol	2.5–500	y = 1771.1x - 19717	0.987	22.375	0.660	1	3
Trehalose	2.5–500	y = 1730.7x - 11099	0.990	23.255	0.686	2	4
Stachyose	2.5–500	y = 1311.1x - 8067	0.992	27.635	0.815	1	3
Raffinose	2.5–500	y = 1342.4x - 9900	0.995	28.817	0.850	1	2
Sucrose	2.5–500	y = 1906.1x - 16636	0.996	29.838	0.880	1	2
Lactose (ISTD)	2.5–500	y = 2054.6x - 34199	0.990	33.920	1	1	2
Cellobiose	2.5–500	y = 1970.3x - 23833	0.993	35.831	1.056	1	2
Galactose	2.5–500	y = 2153.4x - 45884	0.970	38.960	1.149	2	4
Glucose	2.5–500	y = 2229.4x - 49094	0.978	40.294	1.188	2	4
Mannose	2.5–500	y = 1884.7x - 42575	0.983	41.518	1.224	2	4
Fructose	2.5–500	y = 1364.1x - 20967	0.994	42.798	1.261	2	4
Arabinose	2.5–500	y = 1464x - 20100	0.992	44.486	1.311	2	4
Xylose	2.5–500	y = 930.1x - 10477	0.989	48.300	1.424	3	4
Ribose	25–500	y = 1059.4x - 16297	0.950	51.574	1.520	15	25

*Sugars were separated using CZE, with an electrolyte buffer of pH 13.0, at 10 kV, as described in Section “Materials and Methods.” A mix of 15 sugar standards was injected onto the P/ACE MDQ CE system. R-squared values, LOD, and LOQ were calculated as described in Section “Materials and Methods.” Experiment was repeated three times, representative data from one experimental replicate presented here.*

*^a^Retention index = the retention time of each sugar component divided by the retention time of the I-STD; RI = Rt_*sugar*_/Rt_I-*STD*_.*

### Separation of Complex Mixtures of Plant Carbohydrates Using GC–MS

**FIGURE 5:**
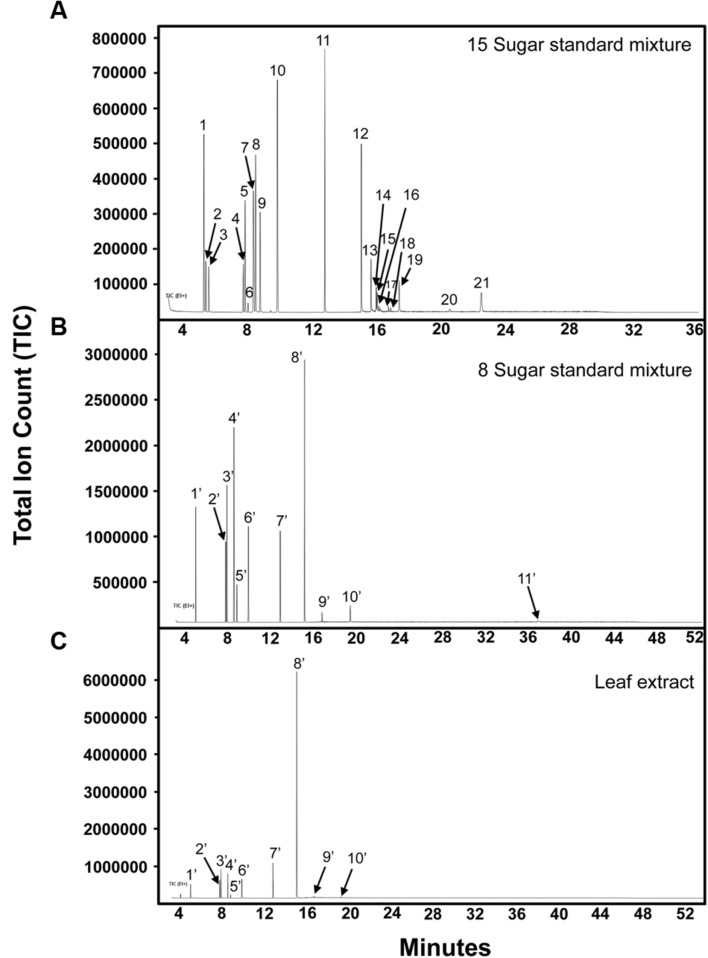
**Separation of soluble sugars by GC–MS. (A)** 15-sugar mixture analyzed at column holding temperature of 300°C. Peak identities: (1) arabinose/xylose; (2) arabinose; (3) ribose; (4,5) fructose; (6) *myo*-inositol; (7) galactose and mannose; (8) glucose; (9) glucose/galactose/mannose; (10) *myo*-inositol; (11) phenyl-β-D-glucopyranoside; (12) sucrose/cellobiose; (13) cellobiose/trehalose; (14) cellobiose; (15) cellobiose/galactinol and lactose (I-STD); (16–19) galactinol; (20) unknown; (21) raffinose. **(B)** Eight-sugar mixture analyzed at column holding temperature of 320°C. Peak identities: (1′) ribitol (I-STD); (2′,3′) fructose; (4′,5′) glucose; (6′) *myo*-inositol; (7′) phenyl-β-D-glucopyranoside; (8′) sucrose; (9′) galactinol; (10′) raffinose; (11′) stachyose. **(C)** Grape leaf extract (75% ethanol extract). Peak identities are the same as described in **(B)**. Experiment repeated three times, representative data shown here.

Gas chromatography–mass spectrometry has traditionally been used in sugar quantification for grape bud and leaf tissues (Grant et al., 2009; Grant and Dami, 2015) as it allows the identification of many soluble sugars with relatively low LOD. In this study, the 15-sugar mixture of both structural and non-structural carbohydrates used in the CZE studies described above (glucose, fructose, sucrose, *myo*-inositol, galactinol, stachyose, raffinose, trehalose, cellobiose, galactose, mannose, arabinose, xylose and ribose, lactose [I-STD]); and a eight-sugar mixture consisting of only non-structural sugars (glucose, fructose, sucrose, *myo*-inositol, galactinol, stachyose, raffinose, and ribitol [I-STD]), were analyzed by GC–MS. As previously published protocols developed for the separation of plant carbohydrates via GC–MS vary in the temperature of the final hold, ranging from 260°C (Long and Chism, 1987; Streeter and Strimbu, 1998) to 320°C (Grant et al., 2009; Grant and Dami, 2015), we choose a “mid-range” final hold temperature of 300°C for our initial analyses. When the 15-sugar mixture was subjected to GC–MS analysis with a final temperature hold at 300°C, most cell wall monomers co-eluted with soluble sugars (i.e., glucose, galactose, and mannose co-eluted, as did sucrose and cellobiose, and cellobiose and galactinol) and the peak for stachyose could not be identified (**Figure 5A**), making it difficult to quantify individual sugars using standard MS-based quantification of specific ions/mass fragmentation patterns. As previously published data (Grant and Dami, 2015) have indicated that higher final hold temperatures help to resolve RFOs in GC–MS analyses, we investigated the degree to which these sugars could be resolved from mono- and di-saccharides using a final hold temperature of 320°C, as described previously (Grant and Dami, 2015). In these assays, an eight-sugar mixture (glucose, fructose, sucrose, *myo*-inositol, galactinol, stachyose, raffinose, and ribitol [I-STD]) was analyzed by GC–MS. Consistent with previously published data (Grant and Dami, 2015), individual peaks for the soluble sugars analyzed were identified (**Figure 5B**) and the peak for stachyose was observed (peak 11). Calibration curves, LOD and LOQ for these soluble sugars using a final hold temperature of 320°C were determined and are shown in **Table 3**. The *R*^2^-values for most soluble sugars were higher than 0.96, with the exception of stachyose (0.946). The LOD and LOQ, of sucrose, galactinol, raffinose, and stachyose were higher in GC–MS analyses (the average LOD and LOQ values for these sugars were 14.3 and 19.8 ng/μL, respectively) than those obtained in the optimized CZE method.

**Table 3:** Linearity, precision, mobility, LOD, and LOQ of sugar components analyzed by GC–MS.

Sugar component	Range (ng/μL)	Calibration curve	*R*-squared	Migration time	Retention index^a^	LOD (ng/μL)	LOQ (ng/μL)
Ribitol^b^	3–400	y = 264.07x - 3383.5	0.981	4.921	0.385	2	3
Fructose	2–800	y = 155.05x - 9294.8	0.991	7.821	0.612	2	4
Glucose	2–800	y = 214.93x - 15468	0.994	8.475	0.663	2	4
*Myo*-inositol	1–350	y = 285.64x - 9191.5	0.966	9.816	0.768	1	2
Phenyl-β-D-glucopyranoside^c^	200	–	–	12.775	1	–	–
Sucrose	2–800	y = 236.22x - 13528	0.988	15.030	1.177	2	4
Galactinol	5–120	y = 101.65x - 428.23	0.994	16.656	1.304	5	10
Raffinose	10–200	y = 83.712x - 1134.2	0.973	19.280	1.509	10	20
Stachyose	40–120	y = 20.4x + 1235.7	0.946	36.750	2.877	40	45

*Sugars were separated using GC–MS, as described in Section “Materials and Methods.” A mix of eight sugar standards was injected into GC–MS system. R-squared values, LOD, and LOQ were calculated as described in Section “Materials and Methods.” Experiment was repeated three times, representative data from one experimental replicate presented here.*

*^a^Retention index = the retention time of each sugar component divided by the retention time of the I-STD; RI = Rt_*sugar*_/Rt_I-*STD*_. For GC–MS samples, phenyl-β-D-glucopyranoside was used as the I-STD for RI calculations.*

*^b^Ribitol was added as I-STD during sugar extraction from grape leaves.*

*^c^Phenyl-β-D-glucopyranoside was added as a second I-STD for GC–MS analyses, as it allows the quantification of the derivatization efficiency.*

### Separation and Quantification of Carbohydrates from Plant Extracts Using CZE vs. GC–MS

As our CZE-based method of sugar quantification seemed to perform comparably to GC–MS analyses on a mixture of sugar standards, we hypothesized that this method would also allow an equivalent, or potentially improved separation of sugars in samples extracted from plant tissues. Given our interest in investigating the role of sugar metabolism in mediating cold stress responses in grape, we used grape leaves as a model tissue for sugar extraction and subsequent quantification. Four extraction solvents, 80, 75, 60% ethanol and water, were evaluated for their ability to extract sugars from grape leaves. Following extraction, CZE-based sugar analyses of leaf extracts were performed using the optimized separation conditions (pH 13.0, 10 kV) described above. No statistically significant differences were observed in either sugar contents or peak resolutions (data not shown) among the different extraction solvents. As the four extraction solvents tested did not show any differences in sugar extraction efficiency in CZE runs, and as it is already established as the extraction solvent of choice for GC–MS analyses of grape tissue (Grant and Dami, 2015), 75% ethanol was selected as the solvent for subsequent sugar extractions from grape leaves.

**FIGURE 6:**
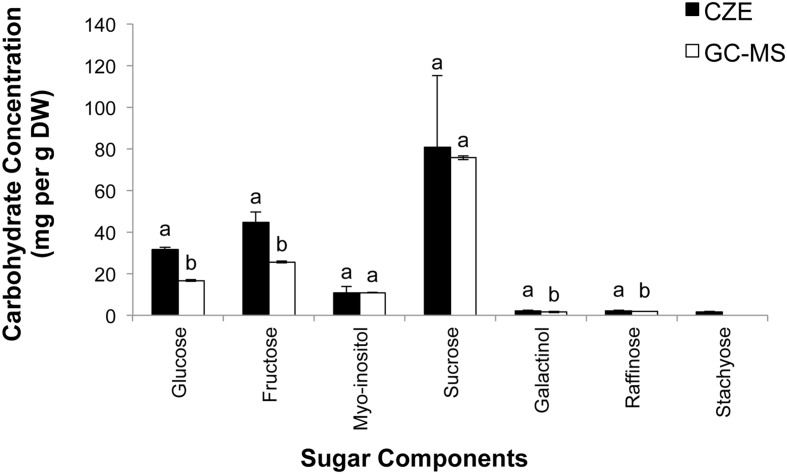
**Quantification of seven soluble sugars in grape leaves using CZE and GC–MS.** Samples of grape leaves were triple-ground in liquid nitrogen, divided in half, and subjected to sugar analysis via either CZE or GC–MS (*n* = 3 technical replicates [i.e., separate extractions of the same leaf sample] for both GC–MS and CZE studies). Values are mean ± SD, letters indicate significant differences according to paired *t*-test, *P* < 0.05.

To compare CZE and GC–MS methods of sugar separation and quantification, a sample of freeze-dried grape leaf tissue was homogenized (see Materials and Methods), and the resulting powders were then divided in half. One half of the sample was analyzed via CZE (**Figure 4B**), while the remaining half was subjected to GC–MS analysis (**Figure 5C**). In our CZE analyses, we were able to successfully detect glucose, fructose, sucrose, raffinose, stachyose, *myo*-inositol, trehalose, galactose, and galactinol in leaf samples. As the peak areas of trehalose and galactose were below the LOQ (see Materials and Methods), we were only able to quantify the contents of glucose, fructose, sucrose, raffinose, stachyose, *myo*-inositol, and galactinol using CZE. Identities of sugars were confirmed by spiking samples with authentic standards and matching retention times and spectral profiles of the observed peaks with those of authentic standards. Surprisingly, in CZE analyses, peaks for most structural sugars (cellobiose, xylose, ribose, arabinose, and mannose) were not observed in leaf extracts. We hypothesized that this was because levels of these sugars were below the LOD achievable by CZE in these samples (**Figure 4B**), and not because the peaks were undetectable due to matrix suppression from the background of the leaf extract. To test this hypothesis, we spiked an additional set of leaf extracts with cellobiose, xylose, ribose, arabinose and mannose standards, and were able to observe these peaks in the leaf sample, indicating that once the concentrations of these sugars crossed the thresholds of LOD and LOQ, they could be visualized and quantified in the background of the leaf extract (data not shown). Since stachyose was only detected in trace amounts via CZE (24 ng/μL), we did not expect to see this peak on the GC–MS trace, as the LOD for stachyose using CZE (1 ng/μL) is considerably lower than the LOD we achieved for this sugar using GC–MS (40 ng/μL; **Table 3**); and indeed no stachyose peak was observed in our GC–MS analyses of leaf extracts. A comparison of the amounts of soluble sugars in leaf extracts, calculated using CZE vs. GC–MS methods, is presented in **Figure 6**. The calculated amounts of sucrose and *myo*-inositol present in samples were not significantly different between the two analytical techniques. Interestingly, the levels of glucose, fructose, raffinose, and galactinol calculated using GC–MS were 89, 75, 23, and 59% lower, respectively, than those calculated using CZE.

## Discussion

Recent studies have established CZE methods to separate and quantify simple sugars (mono- and di-saccharides) found in beverages and fruit juices (Rovio et al., 2007). However, these methods were developed using citrus juices, which have a relatively simple carbohydrate composition consisting almost entirely of three sugars (sucrose, fructose, and glucose; Rovio et al., 2007; Jiang et al., 2015). Similar issues are encountered with CZE methods developed to measure sugars from food samples, as these methods have traditionally been focused on only determining sucrose, glucose, and fructose contents (Montero et al., 2004; Jiang et al., 2015; Toutounji et al., 2015). Because of this, it was unclear the degree to which the methods developed for food and/or fruit juice samples could be used to successfully separate and quantify sugars from more complex plant extracts from vegetative or woody tissues. Plant extracts are expected to contain not only the mono- (glucose and fructose) and di-saccharides (sucrose) commonly found in food and beverage samples (Warren and Adams, 2000), but also mono-, di-, and oligosaccharides involved in base carbon metabolism and plant stress responses (for example, *myo*-inositol, ribose, galactinol, stachyose, and raffinose; Nishizawa et al., 2008; Van den Ende, 2013), and cell wall monomers (i.e., structural sugars: cellobiose, galactose, mannose, arabinose, and xylose; Gibeaut and Carpita, 1994). Previous CZE methods developed for the separation of sugars found in beverages and fruit juices used an alkaline electrolyte buffer of pH 12.6 at 16 kV (Rovio et al., 2007). Under these separation conditions, however, monosaccharides and RFOs such as raffinose and stachyose were only partially separated, and several sugars which serve as cell wall components (i.e., cellobiose, galactose, arabinose, mannose, xylose, and ribose), and which are commonly found in plant extracts, co-eluted with other soluble sugars or were less well-resolved (e.g., fructose and arabinose; **Figure 1A**). Additionally, under these conditions, the peak areas of our standard sugar mix (consisting of 13 sugars commonly found in plant extracts) did not exhibit linear increases in area across the range of concentrations (2.5–500 ng/μL) commonly found in plant extracts (**Table 1**). As plant extracts are expected to contain mono-, di-, and oligosaccharides involved in base carbon metabolism and plant stress responses, as well as structural sugars, resolution of these individual carbohydrate species is essential to accurately quantify sugars extracted from plant tissues. The optimized CZE method developed in this study employed a highly alkaline electrolyte buffer of pH of 13.0 and a separation voltage of 10 kV. As there is an equilibrium between the enediolate form and the aldose and/or ketose form of ionized carbohydrates in aqueous alkaline solution (Honda, 1996; Rovio et al., 2007), the proportion of the enediolate form present in the solution increases as the pH increases (Honda, 1996), which we hypothesize allows for the increased separation of individual sugars that we observed under more alkaline conditions (higher pH). The conjugated carbonyl groups produced in enediolate sugars via β-elimination allow increased UV absorption and, as a result, increased detectability via photo-diode array of ionized carbohydrates (Rovio et al., 2007).

The optimized CZE method performed comparably to previously published GC–MS methods commonly used to profile sugars in grape tissues (**Figure 5C**) and, for several sugars, displayed increased sensitivity. While the GC–MS method investigated failed to separate several non-structural sugars from structural sugars/cell wall components, the CZE-based method allowed resolution of these sugars (for example, separating glucose from galactose and mannose). The CZE method may, therefore, provide the ability to determine the degree to which samples have been contaminated by structural carbohydrates during sugar extractions, and more accurately measure sugar/carbon flux through plant metabolism during stress responses. Most notably, the optimized CZE method, likely due to the high number of theoretical plates provided by this technique, allowed lower LOD and LOQ for most sugar components than were attainable using the GC–MS method. This was particularly true of galactinol, raffinose, and stachyose, which are generally present in relatively low amounts (compared to sucrose, glucose, and fructose) in grape tissues. When measured using CZE, the concentrations of galactinol, raffinose, and stachyose present in grape leaves were higher than when concentrations of these sugars were determined using GC–MS. One reason for this may be the lengthy derivatization process which must be employed prior to analyzing samples via GC–MS. During the derivatization procedure, samples were heated to 70°C and derivatized using HMDS. Following this step, samples were treated with TFA, and any sediment present was the precipitated overnight at 4°C (see Materials and Methods). It is possible that the *O*-glycosidic bonds linking the carbohydrate monomers of galactinol, stachyose, and raffinose were broken during either the heat treatment step of the derivatization process, or by the subsequent addition of the strong acid (TFA), resulting in lower amounts of these sugars being present in derivatized samples. This hypothesis is supported by the fact that high-temperature TFA treatments are commonly used to hydrolyze cell wall polysaccharides into their composite mono- or di-saccharides prior to GC–MS analysis (Gibeaut and Carpita, 1991). Further, the derivatization protocol employed for GC–MS based sample analysis required that any sedimented material resulting from the TFA incubation be removed from samples prior to transfer to target vials and GC–MS analysis. These steps of the derivatization protocol may have resulted in non-specific losses in sugars, as mono-, di-, and polysaccharides may have co-precipitated with other constituents during the TFA incubation. It is therefore possible that the lower values for glucose and fructose that were observed when samples were analyzed via GC–MS are the result of sample losses during the precipitation step, or sample loss which occurred during the additional sample pipetting steps required in the derivatization protocol. The elimination of the sample heating, TFA treatment, and sedimentation steps, as well as the reduction in the number of times that each sample was pipetted or transferred may be why the CZE method was able to achieve lower LOD and LOQ for several sugars than the GC–MS method.

Despite the advantages (i.e., ease of preparation, and relatively low LOD and LOQ) of the CZE method described above, this method does possess several limitations and may not be appropriate for all carbohydrate analyses. For example, the CZE method described here does acquire or make use of mass spectral data, and relies entirely on spectral absorbance for the detection and quantification of sugars. Because of this, the method described here will not allow the identification of unknown sugars (which is better achieved using GC–MS), and requires that sugars of interest resolve relatively well from any unknown sugars present in the sample. Additionally, as the separation of sugars by CZE relies on the separation of molecules based on their charge-to-mass ratio, it is dependent upon the ionization of weakly acidic sugar molecules under alkaline conditions. As a result, sugar polymers with high degrees of polymerization (DP), and therefore very small charge-to-mass ratios, are poorly separated using CZE. When the separation of mixtures of both fructose polymers (inulins) and mannose polymers with high degrees of polymerization (DP ≥ 15 sugar monomers) was attempted using CZE, one large, asymmetrical peak, which contained multiple polysaccharides, was observed in each sample run (data not shown). When the same samples were separated using HPLC coupled to an evaporative light-scattering detector, however, we were able to resolve multiple polysaccharide peaks, which separated based on degree of polymerization (data not shown). The CZE method presented here is therefore not well-suited for the separation and quantification of high-DP polysaccharides, which can only be achieved indirectly using this method. For example, high-DP polysaccharides could by hydrolyzed, either enzymatically or using heat-acid treatments, into their composite mono-, di-, or tri-saccharides, which could then be quantified using the CZE method described above. Polysaccharide levels could then be indirectly calculated based on the concentrations of sugars present in the hydrolysates.

In summary, we have detailed the optimization and validation of a method to quantify sugars from plant tissues using CZE. Our results demonstrate that the optimized CZE method provided equal or greater sensitivity and resolution for most sugars than that achieved by a commonly used GC–MS method. We have successfully used the CZE-based method to quantify sugars from grape leaves, as well as sugars extracted from vegetative and woody tissues of other plant species (data not shown). Since the CZE method described uses very low injection volumes (as low as 19.59 nL per sample [5 s of injection]), it can be used to quantify sugars in very small samples, and we have successfully used this method to quantify sugars extracted from samples as small as 20 mg of tissue. The ability to detect sugars extracted from small amounts of tissue not only simplifies the sample collection process, it also allows the CZE method to be employed in studies designed to rapidly determine sugar contents in plant tissues across developmental stages (e.g., beginning at an early developmental stage, such as pollen, ovules, embryos, etc.; and ending at a later developmental stage like mature seeds, lignified tissues, mature buds, etc.). Finally, both the increased sensitivity and reduced complexity of sample preparation (i.e., no derivatization of samples) make the CZE method described here an ideal system to use in high-throughput metabolomics studies designed to analyze short- and long-term changes in sugar levels involved in plant stress responses.

## Author Contributions

LZ and AC designed and conducted experiments, collected samples, and analyzed data; LZ co-authored the manuscript; NC conducted preliminary experiments; IED maintained grape samples, provided expertise in the role of sugars in cold tolerance, and advised on appropriate grape tissues for carbohydrate analyses; JB designed and supervised experiments, analyzed data, and co-authored the MS.

## Conflict of Interest Statement

The authors declare that the research was conducted in the absence of any commercial or financial relationships that could be construed as a potential conflict of interest.

## Abbreviations

CZE: capillary zone electrophoresis
GC–MS: gas chromatography–mass spectrometry
I-STD: internal standard
LOD: limit of detection
LOQ: limit of quantification
RFOs: raffinose family oligosaccharides
TFA: trifluoroacetic acid.
